# Statins Enhance Clonal Growth of Late Outgrowth Endothelial Progenitors and Increase Myocardial Capillary Density in the Chronically Ischemic Heart

**DOI:** 10.1371/journal.pone.0024868

**Published:** 2011-09-13

**Authors:** Wen Wang, Jennifer K. Lang, Gen Suzuki, John M. Canty, Thomas Cimato

**Affiliations:** 1 Department of Pathophysiology, Capital Medical University, Beijing, China; 2 Department of Medicine/Cardiovascular Medicine, School of Medicine and Biomedical Sciences, SUNY at Buffalo, Buffalo, New York, United States of America; Northwestern University, United States of America

## Abstract

**Background:**

Coronary artery disease and ischemic heart disease are leading causes of heart failure and death. Reduced blood flow to heart tissue leads to decreased heart function and symptoms of heart failure. Therapies to improve heart function in chronic coronary artery disease are important to identify. HMG-CoA reductase inhibitors (statins) are an important therapy for prevention of coronary artery disease, but also have non-cholesterol lowering effects. Our prior work showed that pravastatin improves contractile function in the chronically ischemic heart in pigs. Endothelial progenitor cells are a potential source of new blood vessels in ischemic tissues. While statins are known to increase the number of early outgrowth endothelial progenitor cells, their effects on late outgrowth endothelial progenitor cells (LOEPCs) and capillary density in ischemic heart tissue are not known. We hypothesized that statins exert positive effects on the mobilization and growth of late outgrowth EPCs, and capillary density in ischemic heart tissue.

**Methodology/Principal Findings:**

We determined the effects of statins on the mobilization and growth of late outgrowth endothelial progenitor cells from pigs. We also determined the density of capillaries in myocardial tissue in pigs with chronic myocardial ischemia with or without treatment with pravastatin. Pravastatin therapy resulted in greater than two-fold increase in CD31+ LOEPCs versus untreated animals. Addition of pravastatin or simvastatin to blood mononuclear cells increased the number of LOEPCs greater than three fold in culture. Finally, in animals with chronic myocardial ischemia, pravastatin increased capillary density 46%.

**Conclusions:**

Statins promote the derivation, mobilization, and clonal growth of LOEPCs. Pravastatin therapy in vivo increases myocardial capillary density in chronically ischemic myocardium, providing an in vivo correlate for the effects of statins on LOEPC growth in vitro. Our findings provide evidence that statin therapy can increase the density of capillaries in the chronically ischemic heart.

## Introduction

Coronary artery disease is the leading cause of heart failure in the United States [1]. The primary goal of therapy in patients with heart failure or weakened cardiac muscle function due to coronary artery disease is to restore blood flow to prevent damage to heart muscle from chronic ischemia and prevent death of cardiac myocytes. In some patients coronary artery disease persists unidentified, or cannot be revascularized, resulting in chronically ischemic myocardium, with regional contractile dysfunction. These clinical findings can result in heart failure manifesting as shortness of breath with exertion, weight gain, and retention of fluid. Therapies to promote improved heart muscle function are an important goal to improve symptoms and prevent death in patients with coronary artery disease and heart failure.

Therapy with HMG-CoA reductase inhibitors (statins) is an important component of medical treatment for coronary artery disease primarily for their cholesterol lowering benefits to prevent the progression and recurrence of disease in the heart. However, statins have additional non-cholesterol lowering benefits that are less well described. In our prior study, we found that therapy with the HMG-CoA reductase inhibitor pravastatin mobilized c-kit+ bone marrow cells to the heart in animals with chronic myocardial ischemia with regional myocardial dysfunction, leading to improved contractile function without significantly affecting myocardial blood flow [2]. However, the effects of statins on endothelial progenitor cells and myocardial capillary density in the chronically ischemic heart are not known, and somewhat controversial. Some have suggested that therapy with statins impairs angiogenesis in the chronically ischemic heart [3].

There are several potential sources of new blood vessels in the heart including differentiation of local resident vascular progenitor cells [4] via vasculogenesis, formation of new vessels by recruitment of endothelial progenitor cells from the blood stream, or sprouting angiogenesis of existing blood vessels. Endothelial progenitor cells (EPCs) have been defined in various ways based on cell surface marker phenotypes including CD133 and VEGFR2 expression, or by cultured based assays [5]. However, recent evidence indicates that that an additional cell type contained in blood mononuclear cells known as late outgrowth endothelial progenitor cells (LOEPCs) have distinct growth properties from other EPC cell types, including adherence to collagen coated substrates, the ability to undergo serial replating, clonogenic growth in culture, and the ability to form functional vessels in vivo [6]. Our goal was to determine how HMG-CoA reductase inhibitors affected the mobilization, derivation, and clonogenic growth of LOEPCs as a surrogate for effects of statins on angiogenesis, and determine how statins affect capillary density in animals with chronically ischemic myocardium.

## Materials and Methods

Procedures and protocols conformed to institutional guidelines for the care and use of animals in research.

All our animal work on pigs was performed according to a standing protocol approved by the SUNY at Buffalo School of Medicine and Biomedical Sciences IACUC (Protocol Number MED022011Y). Briefly, all studies where blood was to obtained, or studies on pigs with hibernating myocardium were performed on animals that were sedated using tiletamine (50 mg/mL) and zolazepam (50 mg/mL), followed by intubation and sedation with 0.5–2% isoflurane-oxygen mixture. Terminal procedures were performed using a deep surgical plane of anesthesia prior to euthanasia.

### Preparation of Mononuclear Cells from Porcine Peripheral Blood

Porcine mononuclear cells (MNCs) were isolated as described [7], [8] with minor modifications. Briefly, peripheral blood was collected into cell preparation tubes for density gradient centrifugation (BD Vacutainer CPT, Catalog number 362761), centrifuged for 30 minutes at room temperature, 1500 × g. After centrifugation, the supernatant was discarded, and the mononuclear fraction was retained, washed twice in PBS and resuspended in EBM-2 medium (Lonza, Walkersvile,MD) supplemented with 10% fetal bovine serum (FBS; Hyclone,Logen,UT) (complete EGM-2 medium).

### Culturing of MNCs for Late Outgrowth Endothelial Progenitor Cells (LOEPCs)

MNCs in complete EGM-2 medium were seeded onto six-well tissue culture plates (3×10^7^cells per well) coated with type I rat tail collagen (BD Biosciences, Bedford, MA), and incubated at 37°C with 5% CO_2_ overnight. Nonadherent cells and debris were discarded and adherent cells were washed once with EGM-2 medium, and grown in EGM-2 medium. Medium was changed daily for 7 days and then every other day until first passage. After 7–10 days, monolayers of cobblestone-appearing cells appeared.

### Immunoflurescence Microscopy

Cells were grown on 24 well tissue culture dishes to 80% confluence, and fixed with 4% paraformaldehyde (Electron Microscopy Services Inc.). Cells were blocked in 5% normal donkey serum (Chemicon) for 1 hour. Primary antibody was added (1×100 dilution) in PBS, and incubated with primary antibodies 3 hours to overnight at room temperature. The following primary antibodies were used: Mouse, goat, and rabbit IgG (R&D Systems), CD31; mouse monoclonal, clone LCI-4, Santa Cruz Biotechnology Catalog #SC-80912), CD105; mouse monoclonal, clone MEM-229, Santa Cruz Biotechnology Catalog #SC-51594), CD117; rabbit polyclonal, Santa Cruz Biotechnology Catalog #SC-168), CD144; goat polyclonal, Santa Cruz Biotechnology Catalog # SC-6458), VEGFR2 (Flk-1; mouse monoclonal, clone A-3, Santa Cruz Biotechnology Catalog # SC-6251), eNOS (rabbit polyclonal, Santa Cruz Biotechnology Catalog #SC-654), CD133 (goat polyclonal, Santa Cruz Biotechnology Catalog # SC-19365). Alexa Fluor conjugated 488 or 555 donkey anti-mouse, goat or rabbit secondary antibody (Molecular Probes) was added at a dilution of 1:500 for 1 hour at room temperature. Nuclei were stained with 4′,6-diamidino-2-phenylindole (DAPI). Immunofluorescent images were obtained using a Zeiss Axiovert 200 inverted fluorescence microscope and Zeiss LD Achroplan 10× and 20× objectives using identical exposure times between antibodies. Images were merged using Zeiss Axiovision software.

### Immunophenotyping and Acetylated LDL Uptake by Flow cytometry analysis

Cells were dissociated to a single cell suspension using 0.05% Trypsin-EDTA, resuspended in DMEM/F12 with HEPES supplemented with 10% FBS (Staining Medium), and pelleted by centrifugation at 30 x g. Cells were incubated with primary antibodies for 1 h at 4°C. Porcine specific antibodies used for flow cytometry analysis included the following: CD31 (PECAM) (mouse monoclonal, clone LCI-4, AbD Serotec. Oxford, UK), CD45 (mouse monoclonal, clone K252.1E4, Santa Cruz Biotechnology Catalog # SC-59065), CD105 (Endoglin); mouse monoclonal, clone MEM-229, Santa Cruz Biotechnology Catalog #SC-51594), CD133 (mouse monoclonal, clone AC133, Miltenyi Biotec). Alexa Fluor conjugated 488 donkey anti-mouse antibody (Molecular Probes) was used to detect primary antibodies. Primary antibodies were incubated at 1∶10 dilution, secondary antibodies were incubated at 1∶200 dilution. Uptake of acetylated LDL was determined by incubation of LOEPCs with 10 µg/ml Alexa Fluor conjugated 488 Dil-Ac-LDL (Molecular Probes) in EGM-2 medium 3 hours at 37°C.

Flow cytometry analysis was performed on a BD FACSCalibur. 100,000 events per condition were collected. Flow cytometry data was gated in the FSC vs. SSC plots to exclude dead or clumped cells. Cytometry data were analyzed using FCS Express (DeNovo Software).

### Effects of Statins on In Vivo Mobilization and In Vitro Derivation of LOEPCs from Porcine Peripheral Blood

Peripheral blood was obtained from eight healthy pigs (11–14 weeks old, 40±9 kg, five males, three females) before and after treatment with pravastatin for three days (4 mg/kg orally). Mononuclear cells were isolated using the methods described above. Mononuclear cells were plated at the density of 3×10^7^ cells per well on six-well plates. After culture in EGM-2 medium for 14 days, cells were trypsinized and total cell number per well was determined using a hemocytometer. The percentage of PECAM(+) cells per well were determined by flow cytometry using the protocol described above. The number of PECAM(+) cells per 1×10^7^ blood MNCs plated was determined as the product of total cells per well × percentage of PECAM(+) cells per well. The effect of statin treatment on the in vitro derivation of LOEPCs was determined in MNCs obtained from normal pigs (not treated with pravastatin), and treated with pravastatin or simvastatin (10 nM to 10 µM) added 24 hours after plating.

### LOEPCs Colony-Forming Assays

Early passage LOEPCs (passages 3-5) were seeded onto six-well plates precoated with type I rat tail collagen at clonal density, 500 cells per well or 315 cells/cm2 [9]. The following day, cells were either left untreated (control) or treated with pravastatin (100 nM to 10 µM, Calbiochem, Catalog #524403), simvastatin (100 nM to 10 µM, Calbiochem, Catalog #567021) or Akt inhibitor IV (Calbiochem, Catalog #124015). Seven days after plating, cell colonies were enumerated by phase contrast microscopy at 40x magnification, followed by trypsinization and enumeration of total cells per well.

### Western Blot Analysis

Cells were harvested in homogenization buffer and total protein content was quantified using Bio-Rad's DC Protein Assay. Samples were separated on 10% SDS-PAGE gels, and transferred to PVDF membranes. Blots were probed with the following primary antibodies: Akt (rabbit polyclonal antibody, Cell Signaling Technology Catalog #9272; 1∶1000 dilution, overnight at 4°C), Phospho-Akt (Ser473,736E11, rabbit monoclonal antibody, Cell Signaling Technology Catalog #3787; 1∶1000 dilution, overnight at 4°C), GAPDH (V-18, goat polyclonal antibody, Santa Cruz Biotechnology Catalog #sc-20357; 1∶300 dilution overnight at 4°C). Protein bands were detected by chemiluminescence using horseradish peroxidase conjugated secondary antibodies (WestPico kit, Pierce Biotechnology, 1∶5000 dilution for 1 hour at room temperature), and quantiated by densitometry (Bio-Rad, Quantity One software).

### Matrigel Cord Formation Assay

LOEPCs were plated at a density of 5,000 cells per well in a 96 well tissue culture plates coated with 50 µL of Matrigel (BD Biosciences) per well, and observed up to five days for capillary tube formation. Wells were imaged 48 hours after cells were plated to Matrigel using a Nikon phase contrast microscope and a 2x objective. Images were captured using a Canon EOS digital camera and associated software. Total vessel length and the number of branch points were determined from the captured images using ImageJ. Experiments were performed in triplicate. Significant differences in vessel length and number of branch points with each treatment was determined using an one-way ANOVA with a Holm-Sidak post-hoc test, p<0.05.

### Hibernating Myocardium Model

Hibernating myocardium was produced as previously described [10]. Briefly, pigs were sedated (Telazol; tiletamine 50 mg/mL and zolazepam 50 mg/mL)/xylazine (100 mg/mL, 0.022 mg/kg i.m.), intubated and ventilated with 0.5–2% isoflurane-oxygen mixture. Through a limited pericardiotomy, the proximal LAD was instrumented with a Deltrin occluder (1.5 mm i.d.). Antibiotics (cefazolin, 25 mg/kg and gentamicin, 3 mg/kg i.m.) were given 1-hour before surgery and repeated after closing the chest. Following surgery analgesia was perfomed by intercostal nerve block (0.5% Marcaine), intramuscular doses of butorphanol (2.2 mg/kg q6 h) and flenixin (1–2 mg/kg q.d.).

### Quantitative Immunohistochemistry of Capillary Density

Samples adjacent to LAD (hibernating) and posterior descending arteries (normal) were obtained for immunohistochemical studies. To identify effects on myocardial capillary density, frozen tissue sections (5 µm) were incubated with anti-PECAM1 (clone LCI-4, 1∶100 dilution, AbD Serotec). Images were acquired with a confocal microscope (Bio-Rad MRC 1024). The number of CD31+ cells was determined in 478±43.1 microscopic fields (73.8±3.7 mm^2^).

### Statistical Analysis

Data are expressed as means ± SEM unless otherwise specified.

Statistical comparison between PECAM (+) cell growth from porcine peripheral blood before and after treatment with pravastatin in vivo was performed using paired t-test. All other comparisons were performed using one-way ANOVA; p<0.05 is considered as statistically significant.

## Results

### Derivation and Characterization of Late Outgrowth Endothelial Progenitor Cells from Pigs

**Figure 1:**
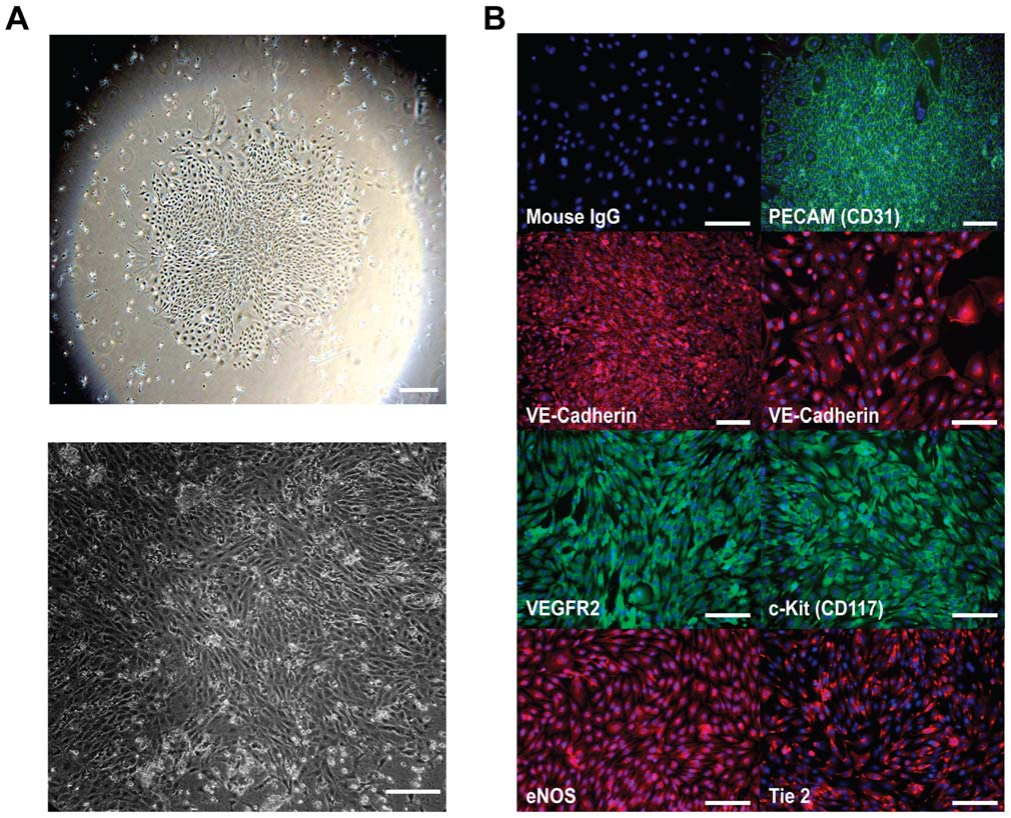
Characterization of porcine late outgrowth endothelial progenitor cells (LOEPCs). **Panel A:** Phase contrast image of porcine LOEPC colony (upper image 2X magnification, lower image 10 x magnification, scale bar 500 microns). **Panel B:** Representative microscopic fields of LOEPCs are shown illustrating antigens characteristic of LOEPCs and endothelial cells; PECAM (CD31), VE-Cadherin (CD144), VEGFR2 (FLK1/KDR), c-Kit (CD117), endothelial specific nitric oxide synthase (eNOS), and Tie 2. Scale bars = 500 um. Results shown are representative of at least three independent experiments from separate animals.

**Figure 2:**
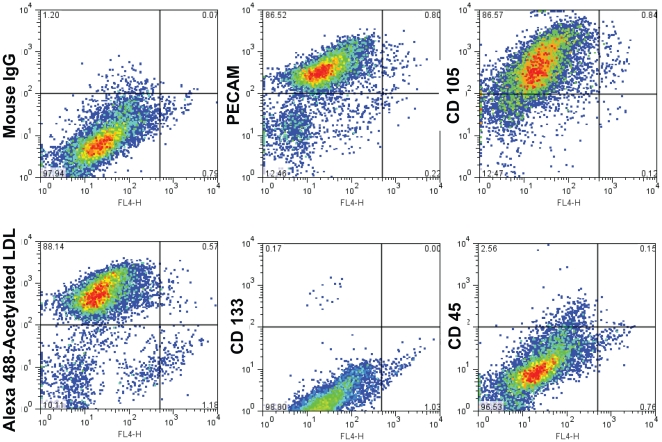
Flow cytometry analysis of porcine late outgrowth endothelial progenitor cells (LOEPCs). Representative flow cytometry plots of cultured late outgrowth endothelial progenitor cells expanded from initial clonal outgrowths from blood mononuclear cells. LOEPCs were analyzed with mouse monoclonal antibodies to the following antigens: Mouse IgG1, Mouse anti-pig CD31, CD105, CD133, and CD45. Primary antibodies were detected with Alexa Fluor 488 conjugated donkey anti-mouse antibodies. Results are representative of at least three independent experiments from separate animals.

We derived LOEPCs from porcine blood mononuclear cells. LOEPC grew as colonies or clones upon initial outgrowth from blood mononuclear cells (Figure 1A), and were passaged serially. Porcine LOEPCs expressed antigens found in endothelial cells, and described in LOEPCs from human blood [7] including PECAM (CD31), VE-Cadherin (CD144), VEGFR2, c-kit (CD117), endothelial specific nitric oxide synthase (eNOS), and Tie 2 (Figure 1B). Additionally, live porcine LOEPCs retained cell surface expression of the endothelial markers CD31, CD105, and absorbed acetylated LDL, but were negative for the early outgrowth EPC marker CD133, and hematopoietic cell marker CD45 (Figure 2). Collectively, porcine LOEPCs have an antigen profile and activities consistent with features found in human LOEPCs.

### Pravastatin Mobilizes LOEPCs into the Blood Stream

**Figure 3:**
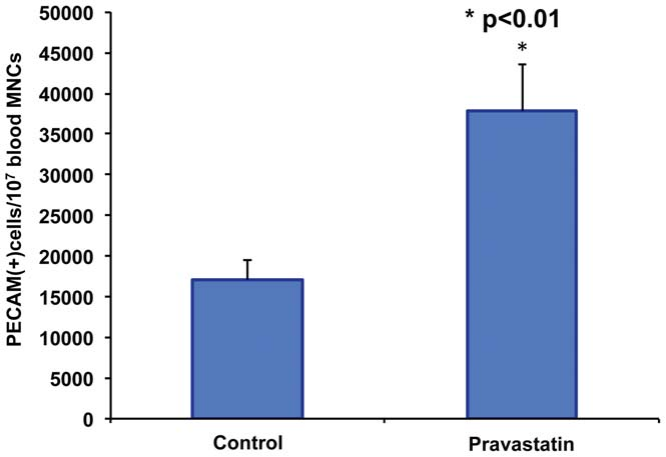
Oral Pravastatin Promotes the Outgrowth of LOEPCs from Blood Mononuclear Cells. Pigs were treated with a placebo or pravastatin (4 mg/kg orally) daily for three consecutive days. Blood mononuclear cells were obtained on day three, and cultured on type I collagen coated dishes to derive LOEPCs. The quantity of LOEPCs per 3×10^7^MNCs plated was determined using flow cytometry to determine the number of PECAM positive cells after 14 days in culture. The data are presented as the means ± SEM from eight different animals. * p<0.01 vs. control using a paired t-test.

Given the positive effects of statins on the quantity and activity of early outgrowth EPCs shown previously, we hypothesized that statin therapy similarly mobilizes LOEPCs into the bloodstream when given to animals in vivo [12]. To test this hypothesis, we collected blood samples from pigs at baseline, and after treatment with pravastatin 160 mg daily for three days in vivo. Blood mononuclear cells were obtained and cultured under conditions that support growth of LOEPCs for 14 days in the absence of further statin treatment. We then determined the quantity of CD31+ LOEPCs per 1×10^7 ^MNCs plated. We found that short-term therapy with pravastatin efficiently mobilizes LOEPCs, increasing the number of LOEPCs greater than two-fold (37,788±5,738 pravastatin vs. 17,050±2,517 untreated, p≤0.05-Figure 3).

### Statins Enhance Derivation of LOEPCs from Mononuclear Cells In Vitro

**Figure 4:**
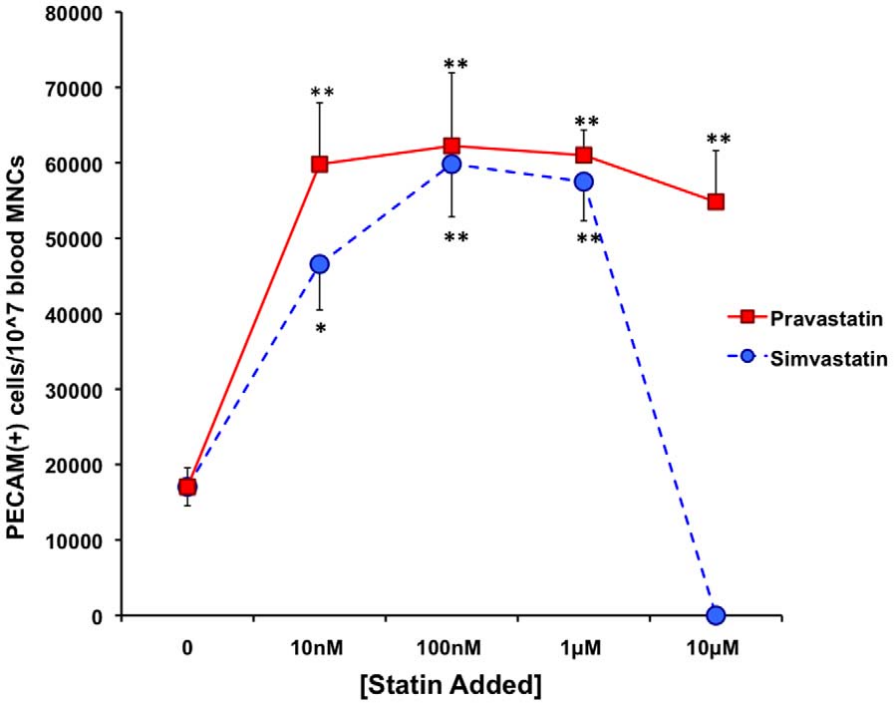
In Vitro Pravastatin and Simvastatin Promotes Outgrowth of LOEPCs from Blood Mononuclear Cells. Mononuclear cells were obtained from normal young pigs and cultured on type I collagen to derived LOEPCs. The quantity of LOEPCs per 3×10^7^MNCs plated was determined using flow cytometry for PECAM positive cells after 14 days in culture. The data are presented as the means ± SEM from eight different animals. * p<0.05, ** p<0.01 vs. control using a paired t-test.

We hypothesized that statin treatment may augment the growth or survival of LOEPCs in culture. To determine the effect of statin therapy on the derivation of LOEPCs, both pravastatin and simvastatin were added to MNCs from normal pigs in vitro, and the quantity of CD31+ cells per 1×10^7^ cells plated was determined by flow cytometry after 14 days of growth. Both pravastatin and simvastatin treatment of normal porcine MNCs in vitro increased the number of LOEPCs obtained greater than three-fold (62,247±9,684 CD31+ cells/1×10^7^ MNCs for pravastatin-100 nM, 59,833±6,999 CD31+ cells/1×10^7^ MNCs for simvastatin-100 nM, 17,050±2,518 CD31+ cells/1×10^7^ MNCs untreated). While higher doses of pravastatin (1 µM to 10 µM) did not have a statistically significant effect on the quantity of LOEPCs obtained, simvastatin at high doses (10 µM) resulted in cytotoxicity (Figure 4). The concentration range of statins added to LOEPCs in our study is also clinically relevant. In normal human subjects, 40 mg of pravastatin taken daily provides a steady state level of pravastatin ∼100 nanomolar in the bloodstream [11].

In conclusion, statin therapy significantly improved the derivation of LOEPCs from MNCs, facilitating their isolation and expansion in culture.

### Statins Augment Clonal Growth of LOEPCs Predominantly by AKT Independent Effects

**Figure 5:**
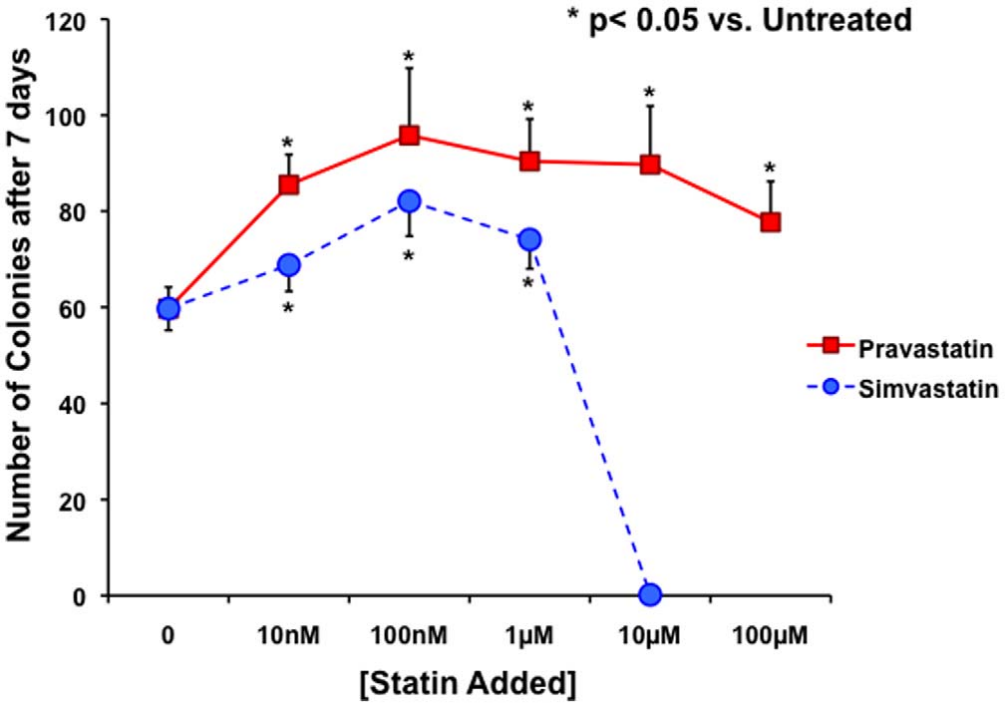
In Vitro Pravastatin and Simvastatin Promotes Growth of LOEPCs under Clonogenic Conditions. LOEPC cell lines from pigs were plated under limiting dilution conditions in the absence or presence of pravastatin or simvastatin. The number of LOEPCs was determined after seven days of growth with or without statin. The data are presented as the means ± SEM from eight independent experiments.

A key difference between LOEPCs and EOEPCs is their ability to grow under limiting dilution or clonal conditions [5], [6]. Hematopoietic cell progenitors are defined by their ability to form colonies based on differences in proliferative or differentiation potential, and the same principal of clonal growth was used to define LOEPCs from humans [7]. Clonogenic growth is a defining assay to distinguish EOEPCs from LOEPCs. The capacity for replating and growth under clonal conditions is a feature specific for the LOEPC and provides additional evidence to support our characterization data [12]. We hypothesized that one effect of pravastatin on LOEPCs was to promote their growth and survival in culture. Additionally, some reports suggested that different statins may have stimulatory vs. inhibitory effects on angiogenesis, but it remains unclear if this effect is true of all statins, or if more subtle chemical differences between compounds may underlie potential differential effects [13], [14]. We compared the effects of the hydrophilic statin pravastatin vs. the hydrophobic statin simvastatin on LOEPC colony growth (see Figure 5). Both pravastatin and simvastatin augmented the growth of LOEPCs with the greatest effect noted at 100 nM. Pravastatin (100 nM) increased the number of LOEPC colonies 60% (95.8±14 colonies formed/500 cells plated vs. 59.7±4.5 for untreated cells, p≤0.05), while simvastatin (100 nM) increased the number of LOEPCs colonies obtained by 38% (82.1±7.3 colonies/500 cells plated). Interestingly, high concentrations of simvastatin (≥10 µM) but not pravastatin resulted in cytotoxicity indicating a differential effect of statin drugs on LOEPCs colony growth.

**Figure 6:**
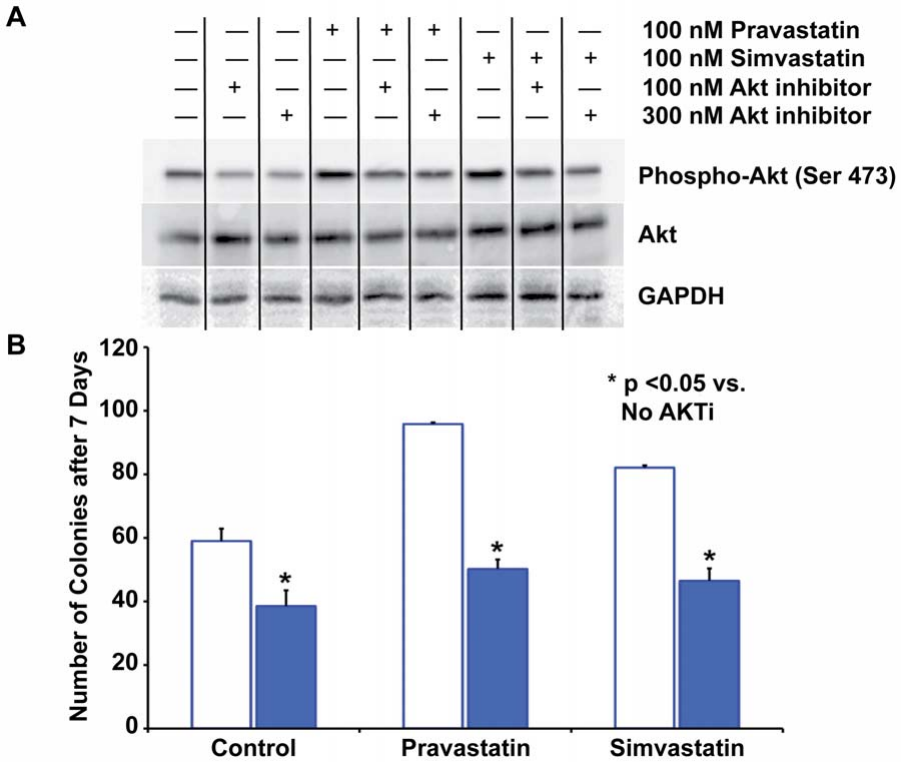
Clonogenic Growth of LOEPCs is Sensitive to AKT inhibition, while the Effect of Pravastatin and Simvastatin on LOEPC Clonogenic Growth is Not. **Panel A-**Western blot analysis showing both pravastatin and simvastatin activate AKT phosphorylation, and AKT inhibitor IV blocks the phosphorylation of AKT in LOEPCs. **Panel B-**LOEPCs were grown under clonogenic conditions with or without an AKT inhibitor (100 nM), in the presence or absence of pravastatin or simvastatin (100 nM each) to determine if the effect of statins on LOEPC clonogenic growth as sensitive to AKT inhibition. n = 6 independent experiments. * p<0.05 vs. No Akt inhibitor IV.

As statins have been shown to mediate their effects on early outgrowth EPCs via the PI-3 kinase/AKT pathway, we asked whether statin treatment of porcine LOEPCs induces AKT phosphorylation [12]. We found that statin treatment in vitro (100 nM) increased AKT phosphorylation 20% when compared with untreated cells (0.823±0.04 densitometric units vs. 1.05±0.08 for pravastatin treated cells, vs. 1.02±0.1 for simvastatin treated cells). We verified the specificity of our western blot detection method using an inhibitor of AKT activity (Figure 6A).

To determine the functional significance of AKT activity on clonal growth of LOEPCs, we treated control and statin exposed cells with an AKT-specific antagonist (AKT inhibitor IV). We used 100 nM AKT inhibitor IV for these experiments because we found higher concentrations resulted in cell death in the majority of the cell cultures. We found that inhibition of AKT activity in LOEPCs reduced the number of colonies formed by 70% (59±3.9 vs. 17.5±3.5 colonies/500 cells plated with AKT inhibitor (100 nM), p≤0.05) in non-statin treated cells, 65% in simvastatin treated cells (82±0.7 vs. 29.2±3.4, p≤0.05), and 63% in pravastatin treated cells (95.8±0.5 vs. 37.3±3.7, p≤0.05, Figure 6B). These findings indicate the majority of the growth and survival properties of LOEPCs are sensitive to AKT inhibition. In contrast to early outgrowth EPCs, statin treatment of LOEPCs in the presence of AKT inhibitor significantly augmented growth relative to untreated controls. These results suggest that statins may act via an AKT independent pathway to promote clonal growth of LOEPCs. The findings support a differential, pleiotropic effect of statins to support the growth and survival of LOEPCs, which may be reflective of statin effects in vivo.

### Effects of HMG-CoA Reductase Inhibitors on Matrigel Tube Formation in Porcine LOEPCs

**Figure 7:**
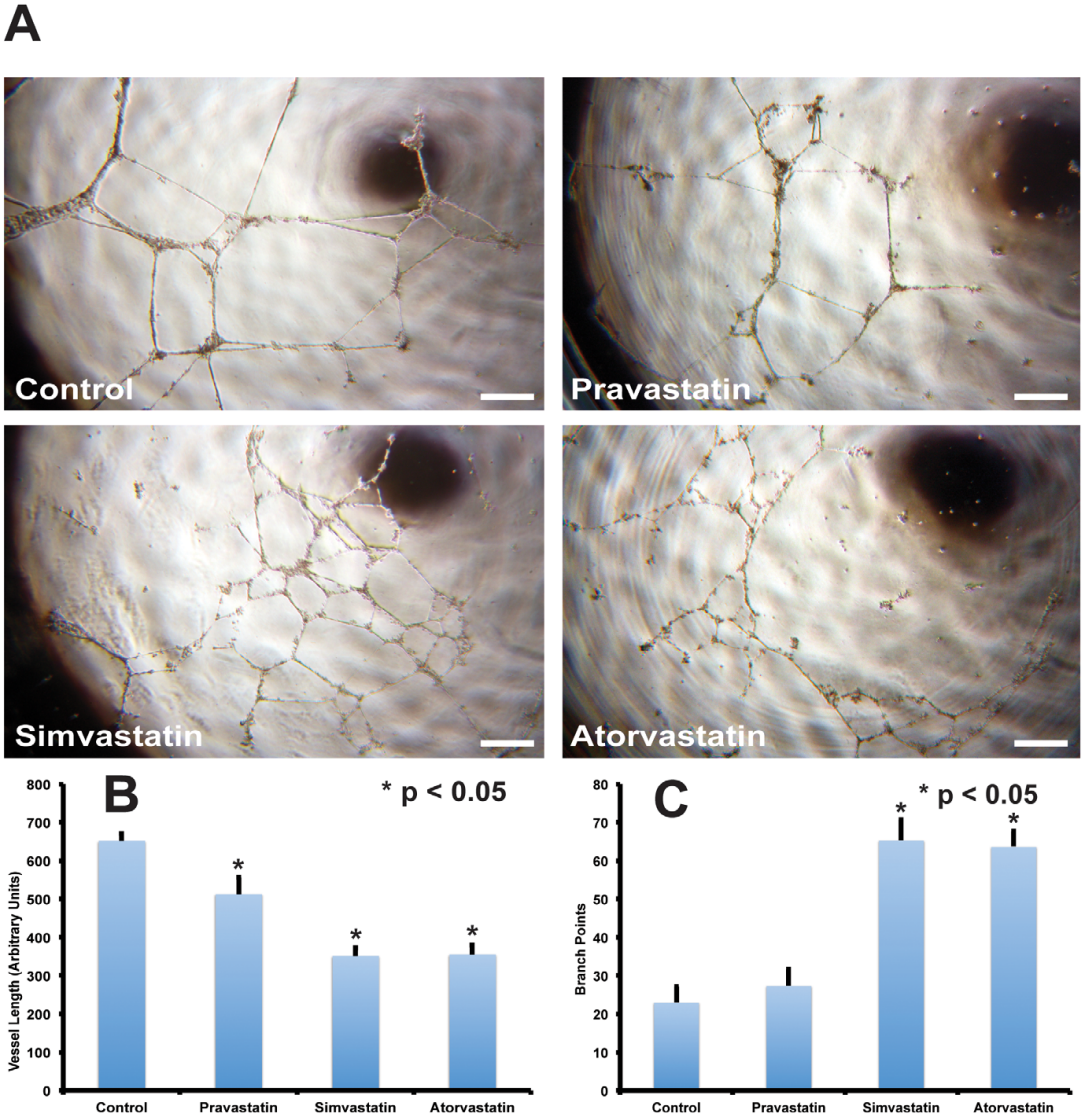
Statins Differentially Affect Angiogenesis of LOEPCs in Vitro. **Panel A:** Representative photomicrographs of porcine LOEPCs plated on polymerized Matrigel for 24 hours with no drug treatment (Control), or Pravastatin, Simvastatin or Atorvastatin (100 nM each). Images are representative of three independent experiments. Scale bar = 500 microns. **Panel B:** Mean vessel length was measured from photomicrographs. Means +/− SEM are shown for three independent experiments. Significant differences from untreated cells (Control) are indicated by * (p<0.05). **Panel C:** Branch Point Determination-the number of branch points within LOEPCs plated on Matrigel was determined from each photomicrograph. The means +/− SEM are shown for three experiments. Significant differences from untreated cells (Control) are indicated by * (p<0.05).

The preceding experiments show that both the HMG-CoA reductase inhibitors Pravastatin and Simvastatin increase the clonal growth of LOEPCs in culture. We next determined the effects of HMG-CoA reductase inhibitors on a different function of endothelial cell biology, formation of tubules in Matrigel. Prior studies have shown that little or no cell proliferation occurs in the Matrigel tube formation assay, but reliably models the process of angiogenic tube formation of endothelial cells in vitro [15]. Therefore the goal of these experiments was to determine if statins differentially affected endothelial cell differentiation and angiogenesis independent of the growth promoting effects of statins [16]. LOEPCs were plated to solidified Matrigel in the absence or presence of Pravastatin, Simvastatin or Atorvastatin (100 nM each). Cells were imaged with phase contrast microscopy 24 hours after plating to Matrigel. Vessel segment length and the number of branch points were determined for each experimental condition. We found that the total segment length of vessels formed in Matrigel was significantly reduced upon incubation with Pravastatin, Simvastatin and Atorvastatin (Figure 7A and B, p<0.05), while Simvastatin and Atorvastatin significantly increased the number of vessel branch points (Figure 7A and C, p<0.05) in comparison with untreated cells. The sum of vessel segments in untreated cells, or after treatment with Pravastatin, Simvastatin, and Atorvastatin was the same in all conditions, consistent with a lack of proliferation of endothelial cells when plated onto Matrigel as previously shown [16]. The findings are consistent with higher potency statins being more effective than Pravastatin in promotion of in vitro angiogenic branch formation in LOEPCs. Moreover, our findings indicate divergent effects of statins on LOEPC clonal growth versus in vitro angiogenesis.

### Pravastatin Therapy Increases Myocardial Capillary Density in Hibernating Myocardium

**Figure 8:**
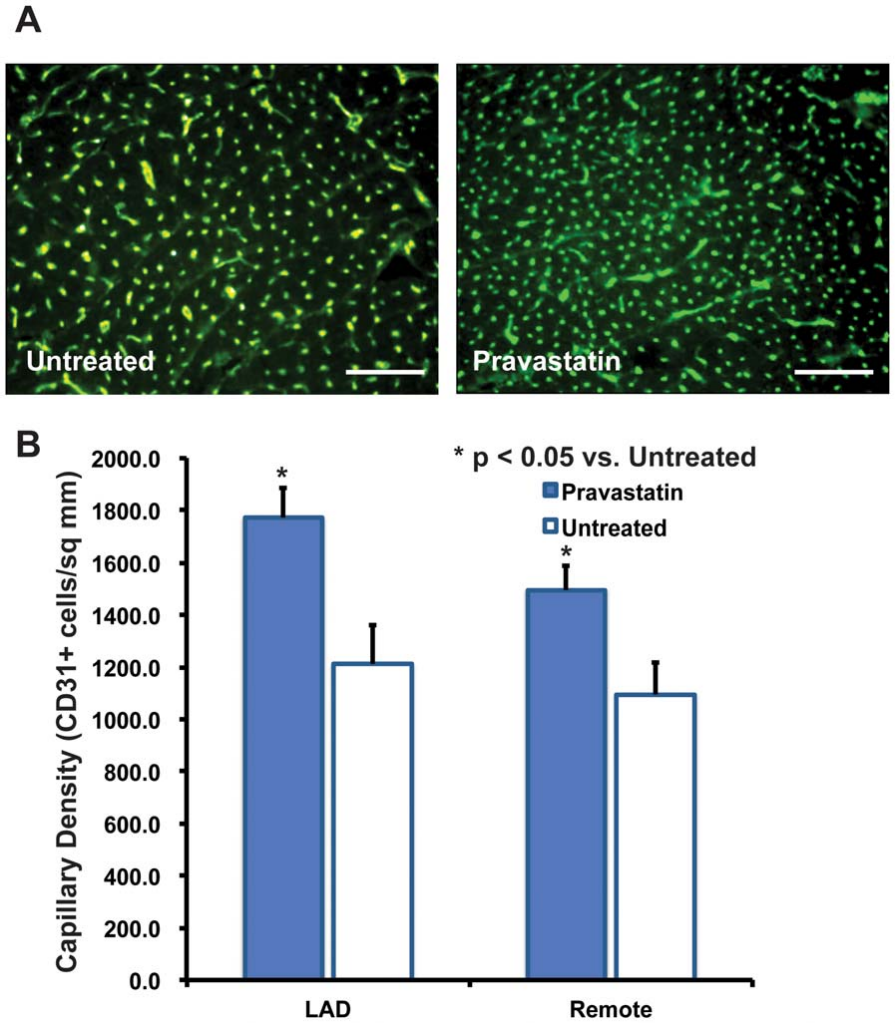
Pravastatin Increases Myocardial Capillary Density in Animals with Chronic Myocardial Ischemia In Both Hibernating Myocardium and the Non-ischemic Remote Myocardium. **Panel A-**Representative immunofluorescence images of myocardial tissue from untreated or Pravastatin treated pigs with hibernating myocardium. Scale bar = 500 microns. **Panel B-**Quantitative assessment of CD31+ cells per square millimeter in chronically ischemic hibernating myocardium in the territory of the left anterior descending artery, and the opposite non-ischemic posterolateral wall, n = 6 animals in pravastatin treated group, n = 5 non-statin treated animals, p<0.05.

To assess the in vivo significance of statin effects on porcine LOEPCs and cardiac angiogenesis, we examined the effect of statin treatment on porcine myocardium. Conflicting studies have suggested that statin therapy may augment or impair angiogenesis in chronically ischemic myocardium [3], [17]. To determine the effect of pravastatin on angiogenesis in the chronically ischemic heart, we measured the capillary density in myocardial tissue obtained from the chronically ischemic anterior wall and non-ischemic posterolateral wall segments from both untreated animals and those treated with pravastatin for 30 days. We found that pravastatin treatment increased the number of CD31+ capillaries per square mm of myocardial tissue by 46% (1769.1±116.7 vs. 1210.6±151.6 capillaries/mm^2^, p≤0.05) in the hibernating LAD territory, and 37% in the remote, non-ischemic territory (1495.2±93.4 vs. 1095.1±123.0, p≤0.05, Figures 8A and B). We have performed similar analysis of CD31+ vessels in the heart using immunoperoxidase labeled secondary antibodies. We found that immunoperoxidase staining did not enhance the visualization of CD31+ cells in the heart. The CD31+ vessels appear small because they are cut in 90° cross section (transversely). The images shown are truly representative of the effect of pravastatin versus no treatment on capillary density in the chronically ischemic heart. The image shown in Figure 8A is representative of one of >400 microscopic fields that were quantified per animal to determine the capillary density. The quantification of the images in panel B reflects the statistically significant difference noted between untreated and pravastatin treated animals.

In previously published work, we found that therapy with pravastatin increased myocyte nuclear density in the chronically ischemic heart with proliferation of small cardiac myocytes [2]. These findings are consistent with an effect of pravastatin that increases the myocardial capillary density to match the microcirculation with formation of new and smaller cardiac myocytes.

## Discussion

From these studies we identified the following novel conclusions: 1) LOEPCs are effectively mobilized into the blood after short-term therapy with pravastatin, 2) both pravastatin and simvastatin facilitate the derivation of LOEPCs from peripheral blood mononuclear cells, 3) low concentrations of both pravastatin and simvastatin enhance clonogenic growth of LOEPCs in vitro, while higher concentrations of simvastatin have toxic effects, 4) antagonism of AKT signaling impairs clonogenic growth of LOEPCs, but does not impair the positive effect of statins on LOEPC growth, 5) in vivo pravastatin increases capillary density in chronically ischemic myocardium in parallel with increased myocyte nuclear density.

### The Relationship Between Early and Late Outgrowth EPCs

The identification and measurement of EPCs has evolved considerably since their original description by Ashahara et al. [18]. However, LOEPCs lack specific markers to identify the cells de novo and quantification of EPCs relies on culture-based assays. Differences in adherence to either fibronectin or type I collagen coated dishes distinguish early and late outgrowth EPCs growth, respectively. Early outgrowth EPCs are functionally distinct from LOEPCs in that they are unable to proliferate under clonogenic conditions, express cell surface markers consistent with monocytes, and fail to form functional vessels when transplanted in vivo [5], [6]. While the relationship between early outgrowth EPCs and cardiovascular disease risk factors has been established [19], the relationship between cardiovascular disease risk and LOEPC growth remain to be defined. Given the fact that LOEPCs proliferate in culture, this trait facilitates studies of the effects of aging of LOEPC growth and activity while EOEPCs cannot be studied in this manner. Furthermore, the clonogenic growth assay for LOEPCs may be applied as we have used it to study effects of statins to identify novel agents that promote their growth and survival in vitro, that may then be translated angiogenesis in vivo.

### The Origins of Late Outgrowth EPCs

Early outgrowth EPCs are identified by expression of CD34, VEGFR2 and CD133. Recent evidence indicates that these cells also express CD45 (indicating a leukocyte cell type) and cell sorting experiments have illustrated that these cells are a hematopoietic progenitor and form monocytes in vitro [20]. Additionally, cell sorting experiments of mononuclear cells from umbilical cord and adult blood has shown that LOEPCs can be obtained from CD34+ VEGFR2+ CD45- and CD133- cell populations, while CD34+ CD45+ cells also express CD133, and generate monocytic cells that have properties consistent with early outgrowth EPCs [20].

Since late outgrowth EPCs lack specific identifying cell surface markers, genetic marking to facilitate fate mapping studies in vivo are currently not feasible. It is plausible that LOEPCs are derived from the bone marrow, or they arise directly from the vessel wall [21]. Endothelial cells with hemogenic potential have been described in the embryonic yolk sac, dorsal aorta, and fetal liver that originate from the vessel walls [22]. Yolk sac derived hemangioblasts, bi-potential cells producing both hematopoietic and endothelial cells have been described in vitro from embryonic stem cells [23]. In vivo fate mapping experiments indicate that multiple cellular sources contribute to yolk sac blood islands in development [24]. LOEPCs in adults may similarly be derived from a polyclonal origin. Specific identifying features to track the fate of LOEPCs in vivo are required to determine their contribution to vessel homeostasis under both normal conditions and in atherosclerotic vascular disease, as well as neoplastic and ischemic angiogenesis.

### Statin Potency and Dose Dependence on Late Outgrowth EPC Growth, Relevance to Angiogenesis In Vivo

Statins are recommended therapy for the primary and secondary prevention of coronary artery disease [25], [26], [27]. Most recent guidelines advocate treating to a target LDL cholesterol <70 mg/dL, because of the effect on cardiovascular disease outcomes in large clinical trials [27], [28]. However, several groups have reported an apparent biphasic or differential effect of statins, in that low concentrations of statins appear to promote endothelial cell proliferation, migration and differentiation, while higher doses impair these effects [3], [17], [29]. One mechanism may be that lower dose statin therapy may activate AKT signaling, but impair the function of isoprenylated proteins of the Ras family or lamins, which have essential functions in cell proliferation and division [30]. An alternative hypothesis would be that the effect of statins on angiogenesis is not tied to statin dose necessarily, but to differences in lipophillicity [31] and the intracellular concentration of statins. In our study, we found effects of pravastatin that promoted clonogenic growth of LOEPCs (10 µM), while the same dose of simvastatin had toxic effects on the cells. The effects of statins in this cell based assay cannot alter lipoprotein levels (i.e. low density lipoprotein), but may function through direct effects on cholesterol biosynthesis affecting lipid modifications of prenylated proteins. This suggests the difference in chemical properties of these two statins may underlie the differences in LOEPC growth and survival.

Our findings show that statins increase both the derivation of LOEPCs from blood and growth under clonogenic conditions. While AKT activity plays a role in their clonogenic growth, antagonism of AKT does not completely inhibit the effect of statins to promote LOEPC growth. Additionally, in the chronically ischemic heart, we find that pravastatin therapy increases the myocardial capillary density in both chronically ischemic myocardium, and the less affected remote myocardial territory. These findings indicate that both in vitro and in vivo statins promote the growth of nascent endothelial cells. Additionally, recent evidence suggests that intracoronary injection of LOEPCs in pigs that have sustained a myocardial infarction may have a beneficial effect on heart remodeling as compared with similar therapy with mesenchymal stem cells [32], suggesting a potential role for LOEPC therapy in animal models and patients with acute myocardial infarcts. LOEPCs may have an advantage over other adult stem cell therapies for acute heart disease including mesenchymal stem cells or cardiosphere derived cells in that they can readily obtained from venous blood. This application of LOEPCs adds significance to our findings that LOEPCs can be expanded in vitro by the addition of pravastatin.

### Study Limitations

Our study shows that LOEPCs can be derived from young pigs and have markers, activity and growth properties that match LOEPCs derived from humans. The success of these studies may be due to the age of the animals used in this study. Remarkably, LOEPCs in humans have both low and high proliferative potential, and cells derived from adults predominantly have low proliferative potential. Though not addressed directly in the current manuscript, we found no difficulties in repeated passaging of LOEPCs to suggest they developed senescence. Similar studies of the effects of statins in LOEPCs from humans would be informative to determine if the findings hold true across species. Furthermore, it is currently difficult to know the precise role of the LOEPC in formation of new vessels in the ischemic heart or other organs, as a lack of cell specific markers for LOEPCs exist to perform in vivo cell fate tracking.
